# *Gardnerella* Revisited: Species Heterogeneity, Virulence Factors, Mucosal Immune Responses, and Contributions to Bacterial Vaginosis

**DOI:** 10.1128/iai.00390-22

**Published:** 2023-04-18

**Authors:** Elinor Shvartsman, Janet E. Hill, Paul Sandstrom, Kelly S. MacDonald

**Affiliations:** a Department of Medical Microbiology and Infectious Disease, University of Manitoba, Winnipeg, Manitoba, Canada; b JC Wilt Infectious Diseases Research Centre, Public Health Agency of Canada, Winnipeg, Manitoba, Canada; c Department of Veterinary Microbiology, University of Saskatchewan, Saskatoon, Saskatchewan, Canada; d Department of Internal Medicine, University of Manitoba, Winnipeg, Manitoba, Canada; University of Pittsburgh

**Keywords:** *Gardnerella*, bacterial vaginosis, vaginal microbiome, sialidase, vaginolysin, cytokines, mucosal immunity, biofilms, chemokines, dendritic cells, female reproductive tract

## Abstract

*Gardnerella* species are associated with bacterial vaginosis (BV) and have been investigated as etiological agents of the condition. Nonetheless, the isolation of this taxon from healthy individuals has raised important questions regarding its etiological role. Recently, using advanced molecular approaches, the *Gardnerella* genus was expanded to include several different species that exhibit differences in virulence potential. Understanding the significance of these different species with respect to mucosal immunity and the pathogenesis and complications of BV could be crucial to solving the BV enigma. Here, we review key findings regarding the unique genetic and phenotypic diversity within this genus, virulence factors, and effects on mucosal immunity as they stand. We also comment on the relevance of these findings to the proposed role of *Gardnerella* in BV pathogenesis and in reproductive health and identify key gaps in knowledge that should be explored in the future.

## INTRODUCTION

**FIG 1:**
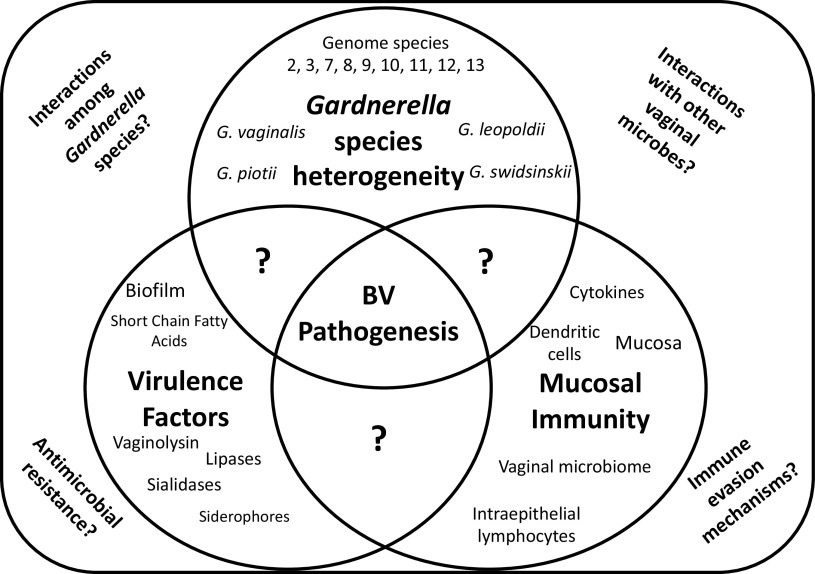
Key gaps in knowledge with respect to *Gardnerella* heterogeneity and contributions to the vaginal microbiome and to BV pathogenesis.

The cervicovaginal microbiome profoundly shapes reproductive health (1). The current consensus asserts that an optimal cervicovaginal microbiome in reproductive-age individuals is dominated by L. crispatus and several other non-*iners* lactobacilli (2). These lactobacilli confer protection from infections via the production of lactic acid and other antimicrobial products (3). Though several nonoptimal cervicovaginal microbial communities have been associated with clinically defined conditions, such as aerobic vaginitis (4) and vulvovaginal candidiasis (5), bacterial vaginosis (BV) is the most common one in reproductive-age individuals (6). During BV, the vaginal microbiome is characterized by a drastic reduction or complete lack of vaginal lactobacilli and increased abundance and quantities of anaerobic and facultative bacteria, whether examined by phenotypic methods such as Gram stain and light microscopy or by molecular methods using both compositional and quantitative genomics (2, 7–9). In addition, BV has also been linked to several reproductive sequelae, including pregnancy complications and infertility (10, 11), as well as increased susceptibility to sexually transmitted infections (STI), including increased acquisition risk and transmission of human immunodeficiency virus (HIV) (12, 13). The mechanistic link between BV and these associated sequelae remains unclear, though it potentially involves alterations in host mucosal immunity (14). Gardnerella vaginalis has been proposed as an etiological agent of BV as early as the 1950s (15, 16), although the isolation of this taxon from healthy individuals has raised questions regarding its etiological role (15, 17, 18). A critical review of *Gardnerella* as a cause of BV was recently published (15). More recently, the description of *G. vaginalis* was modified to include four species and multiple genomospecies (19). This has sparked new research questions regarding the clinical and immunological relevance of these now distinguishable taxa (Fig. 1). Here, we review how the molecular heterogeneity of *Gardnerella* species reflects differences in virulence potential and host immune responses to better understand their contributions to reproductive health.

## *GARDNERELLA* HETEROGENEITY — BEYOND “JUST GENETICS”

The *Gardnerella* genus of *Actinobacteria* (which have a high G-C content), includes several thin-walled Gram-positive pleomorphic species (20, 21), that until recently were collectively classified as *G. vaginalis*. The first *Gardnerella* isolate was isolated by Leopold in 1953 on modified blood agar from cervical swabs and urine samples (22). Within the following 2 years, two reports by Gardner and Duke described the same bacterium as a proposed agent of BV (nonspecific vaginitis) (16, 23). Initial efforts to differentiate pathogenic and nonpathogenic *Gardnerella* strains used biotyping based on biochemical tests. Eight *Gardnerella* biotypes were described based on hippurate hydrolysis, lipase activity, and beta-galactosidase activity (24). Additional *Gardnerella* biotypes were resolved through added biochemical characterization (25–27). Although certain biotypes (e.g., lipase-positive) appeared to be somewhat associated with BV (28, 29), this was not consistent across studies (30). Additionally, no clear associations were identified between these biotypes and known bacterial virulence factors (31–33). The clinical relevance of these biotypes therefore remains unclear, and the use of this system for the study of *Gardnerella* isolates has mostly been supplanted by molecular methods.

Earlier molecular attempts to decipher the genetic heterogeneity of *Gardnerella*, involved the use of restriction fragment length polymorphism. This revealed several genotypes but without a clear clinical relevance (34, 35). The description and taxonomy of *G. vaginalis* has since been changed following the description of four genetically distinct clades/subgroups identified by whole-genome sequencing and *cpn*60 barcode sequencing, and further species designation has been added and confirmed with subsequent molecular characterization (19, 36–38). The following *Gardnerella* species names along with several genomospecies have now been adopted: *G. swidsinskii* and *G. leopoldii* (subgroup A/clade 4), *G. piotii* (subgroup B/clade 2), *G. vaginalis* (subgroup C/clade 1), and subgroup D/clade 3 members, which include several genomospecies (19, 39) (Table 1). Future designation of additional *Gardnerella* species is likely with the genomic characterization of additional isolates. The expanded description of subgroup A to include two distinct species, *G. leopoldii* and *G. swidsinskii*, is somewhat supported by observations that these species do not tend to cooccur more often than other *Gardnerella* species (39). These genetically related though distinct genomospecies, are also reliably distinguished using matrix-assisted laser desorption/ionization time of flight mass spectrometry, which may be relevant for clinical tests and future studies of the cervicovaginal microbiome and BV (19). Structural and morphological differences also exist among these newly described *Gardnerella* species. For example, a *G. swidsinskii* isolate was found to possess a polysaccharide-like capsule, which was absent in a *G. leopoldii* isolate (40). Importantly, these structural variations may elicit different immune responses by stimulating different toll-like receptors (TLR) and other pattern recognition receptors (PRR), so further study is important (41, 42). Although 16S rRNA genes microbial profiling is the most commonly used method for the study of the vaginal microbiome, it does not generally discriminate different *Gardnerella* species (39). This is likely due to a combination of factors, including the presence of multiple copies of this genetic target within a single genome and a reduced barcoding gap compared to protein-encoding gene targets such as *cpn*60 (43).

**TABLE 1:** *Gardnerella* spp. nomenclature and classification of common isolates, indicating key virulence factors^*a*^

Species designation	Subgroup/clade	Ecotype	Vaginolysin type (if present)	Sialidase activity (of tested isolates)	Known isolates
*G. vaginalis* genome sp. 1	C/1	1A, 1B (JCP7275)	1A + 1B	−/+	ATCC 14019, ATCC 14018, ATCC 49145*, JCP7276, UGent 25.49*, HMP9231, UGent 09.01*, 3549624*, 284V, UGent 09.07*, 75712, 315-A, JCP7672, 0288E, JCP7275
*Gardnerella sp.* genome sp. 2	C/1	1B	1B	−	JCP8108, 1400E, 55152, 41V
*Gardnerella sp.* genome sp. 3	B/2	2B	1A + 1B	−/+	GED7275B*, JCP8017A, JCP8017B, JCP7659, 00703C2mash, JCP7719
*G. piotii* genome sp. 4	B/2	2A	1B	+	JCP8151A, JCP8151B, JCP8522, JCP8070, JCP8066, UGent 21.28*, UGent 18.01*
*G. leopoldii* genome sp. 5	A/4	3B	2	−	AMD, UGent 06.41*, UGent 09.48*, 6420B
*G. swidsinskii* genome sp. 6	A/4	3B	2	−	5-1, GS 9838-1*, GS 10234*, GV37*, 409-05
*Gardnerella sp.* genome sp. 7	A/4	3A	1B + 1C	−	JCP8481A, JCP8481B, PSS_7772B*
*Gardnerella sp.* genome sp. 8	D/3	3A	1B	−	101, 00703Dmash
*Gardnerella sp.* genome sp. 9	D/3	3A	1A	−	6119V5
*Gardnerella sp.* genome sp. 10	D/3	3A	1B	−	1500E
*Gardnerella sp.* genome sp. 11	B/2	−	−	+	GED7760B
*Gardnerella sp.* genome sp. 12	Unassigned/broadly A/4	−	3	−	CMW7778B
*Gardnerella sp.* genome sp. 13	Does not fit into the defined subgroups/clades	−	1B	−	KA00225
Ref.					
(19)	(36, 37)	(45)	(95)	(19, 104, 111)	(39, 159)

a −, unknown; −/+, sialidase activity confirmed in some isolates (*in vitro*), but not in others; +, (*in vitro*) confirmed sialidase activity; *, ecotype designation not available.

Ecotypes are genetically related strains that are otherwise ecologically distinct from others (i.e., possess different ecological roles) (44, 45). Currently proposed hypotheses speculate that different *Gardnerella* strains possess different ecological roles in BV pathogenesis (46). In line with this, three major *Gardnerella* ecotypes have been identified so far (using 35 draft and complete genomic sequences), roughly corresponding with the four clade/subgroup descriptions with a few exceptions (Table 1) (45). Ecotype 1 strains possess enriched genetic material related to pentose interconversion, galactose metabolism, and ATP-binding cassette transporters, ecotype 2 strains possess two distinct sialidase encoding genes (among other features), and ecotype 3 encompasses the rest of the species which display underrepresentation of the above genes and overabundance of other unique factors such as metalloendopeptidase related membrane proteins (45). Further characterization of these ecotypes, and expansion of the analysis to include more *Gardnerella* isolates and description of the ecotypes of other BV-associated bacteria could be useful for deciphering their contributions to BV.

*In vitro* studies of different *Gardnerella* isolates (belonging to different subgroups/clades) have provided insight to both phenotypic differences and species interactions. *In vitro*, subgroups A, B, and C thrive the most when grown as single/monococultured isolates, whereas subgroup D isolates have been observed to increase their growth rate as the number of competitors increases (47). This finding is congruent with *cpn*60 subgroup or clade-based characterizations of vaginal microbial communities where *Gardnerella* subgroups A, B, and C tend to dominate communities and reach high bacterial loads, whereas subgroup D is not generally a very abundant taxon and tends to occur more frequently alongside the other clades or other taxa (48–52). This may be due to the ability of subgroup D isolates to utilize a wider range of carbon sources relative to the other *Gardnerella* subgroups, acting as nutritional generalists, which promotes their growth in the vaginal microbiome through negative frequency dependent selection (53).

Regardless of BV status, subgroup A species are commonly detected at a higher frequency in the vagina compared to the other species (48, 52, 54). Nonetheless, there is no clear consensus regarding Nugent-BV and *Gardnerella* species associations, possibly due to differences in study populations. For example, a study using a cohort of nonpregnant premenopausal women found clades 1 and 3 were associated with Nugent-BV, whereas clades 4 and 2 did not exhibit these associations; notably clade 2 was found to associate with intermediate Nugent scores (54). In another cohort of women reporting a female sexual partner, clades 1, 2, 3, and microbial communities with multiple clades were associated with BV (52). Using the newer species designation, increased relative abundances of *G. piotii*, *G. vaginalis*, and *G. swidsinskii* were shown to be associated with Nugent-diagnosed BV in vaginal samples from Canadian women (39). In that same study, increased relative abundances of *G. vaginalis* and *G. swidsinskii* were associated with the presence of abnormal discharge and vaginal malodor, whereas the other two species were not (39). Whether these associations occur in other populations remains to be determined. With respect to treatment outcomes, *G. leopoldii*/*G. swidsinskii* (the primers used did not differentiate between the two) were more abundant among those with recurrent BV, whereas high abundance of genomospecies 7 was associated with a refractory response following oral metronidazole treatment (55). *In vitro*, clades 3 and 4 are inherently resistant to metronidazole, which could help explain these findings (56). However, in a study of premenopausal individuals with BV, decreases in clade 4 and 1 (measured by qPCR) posttreatment were observed regardless of clinical outcome (57).

## VIRULENCE FACTORS AND PROPOSED ROLES IN BV PATHOGENESIS

Several hypotheses have been proposed regarding the contribution of *Gardnerella* species to BV. One hypothesis is that every *Gardnerella* species has the potential to cause BV, while others propose certain *Gardnerella* strains are genetically driven toward a more pathogenic phenotype (46). In line with these arguments, both BV- and non-BV associated *Gardnerella* isolates show evidence of horizontal gene transfer, and evidence of adherence and cytotoxicity of epithelial cells, albeit BV-associated *Gardnerella* isolates appear to adhere and cause cytotoxicity somewhat more efficiently (40, 58, 59). *Gardnerella* species possess several virulence factors, including a hemolysin, mucus degrading sialidases, and the ability to form biofilms which contribute to their “pathogenic” phenotype. The contributions of each of these different virulence factors have been examined in detail using *in vitro* and *in-silico* approaches.

### *Gardnerella* biofilms.

BV is commonly associated with the presence of vaginal squamous epithelial cells studded with bacteria (“clue cells”) that are typically dominated by *Gardnerella* (16, 60, 61). The importance of *Gardnerella* to this BV-biofilm structure was not fully recognized until 2005 (62). Interestingly, evidence exists of sexual transmission of *Gardnerella* biofilms but not dispersed *Gardnerella* forms (63), which may, in part, explain why some individuals colonized by *Gardnerella* do not develop BV or transmit it to others. This finding even prompted a proposal to refer to the biofilm form as “gardnerellosis” (63). *Gardnerella* species have been shown to be superior at biofilm formation compared to other BV-associated bacteria (64), although isolate-specific differences have been noted *in vitro* (65). Coaggregation or the attachment of bacteria to one another facilitates biofilm attachment and was shown to occur between other BV-associated bacteria and *Gardnerella* (66). Little is known with respect to how *Gardnerella* initiates biofilm formation *in vivo*, though this process likely involves bacterial lectins such as serine-rich-repeat adhesins, which have been predicted in several *Gardnerella* isolates (67). These carbohydrate-binding proteins can attach to glycosylated components in the mucosa, forming a “two-way” host-*Gardnerella* interaction that favors bacterial biofilm proliferation. In addition, a collagen-binding Cna protein was identified on the surface of a *G. vaginalis* isolate (68) and may also play a role in biofilm establishment and potentially in immune evasion via interactions with complement proteins (69). Further characterization of these interactions will be necessary to explore the potential of these carbohydrate-binding and collagen-binding proteins as therapeutic targets or for diagnostics.

Once formed, bacterial biofilms are characterized as aggregated bacterial cells embedded in a sticky extracellular matrix (ECM). The composition of an ECM varies among organisms, but generally consists of macromolecules, and predominantly exopolysaccharides (70). Unsurprisingly, in a *G. leopoldii* isolate, transcripts associated with adhesion and exopolysaccharide biosynthesis such as glycosyltransferases were upregulated in biofilms compared to planktonic counterparts (71). These *Gardnerella* biofilms exhibit increased resistance to metronidazole (72) and clindamycin treatment (73), and reduced sensitivity to antibacterial metabolites such as hydrogen peroxide and lactic acid (74). A study of a single *Gardnerella* (subgroup C) isolate showed its biofilm contains a higher carbohydrate content compared to planktonic cultures, with relatively similar nucleic acid and protein content (74). *N*-acetylglucosamine appears to be an important contributor to this *Gardnerella* isolate biofilm ECM, although chitinase treatment did not significantly reduce biofilm thickness (74). *In vitro*, proteinase treatments appear to reduce *Gardnerella* biofilms and increase their susceptibility to antimicrobials, possibly due to degradation of bacterial anchor proteins (72, 74).

Extracellular DNA (eDNA) is another important component of bacterial biofilms thought to be released from lysed cells. Degradation of eDNA by DNases appears to inhibit biofilm formation and reduce biofilm density *in vitro* and decrease (but not eliminate) *Gardnerella* colonization in a murine model (75). These findings suggest eDNA is vital for *Gardnerella* biofilm formation and structural integrity, though questions remain regarding where the eDNA is localized within the biofilm, especially *in vivo*. The contributions (if any) of the host to the biofilm eDNA have not yet been explored either, though given the increases in exfoliated epithelial cells in BV samples (76), this cellular turnover could be a potential contributor, as was previously demonstrated in the gut mucosa (77). ECM composition also affects immune recognition (78). A transcriptomics study of a *G. leopoldii* isolate showed that at least *in vitro*, the biofilms exhibit reduced transcription of metabolic and ribosomal genes suggestive of reduced metabolic activity (especially carbon metabolism) (71). This could explain the observed resistance to antibiotics such as metronidazole (56). Metronidazole enters bacterial cells as a prodrug that is reduced intracellularly to an active form (79), and thus reduced metabolic activity reduces activation of metronidazole. Transcripts associated with protein export and amino acid biosynthesis were also highly expressed in these biofilm cultures (71). Amino acids produced by *Gardnerella* can be utilized mutualistically by *Prevotella* species and may thus contribute to the growth of other BV-associated bacteria within the biofilm structure (80, 81). BV-associated species can then metabolize the *Gardnerella* produced amino acids to biogenic amines which are the likely source of malodor during BV (82). Whether or not amino acid metabolism is upregulated in all *Gardnerella* species biofilms is unclear, although it could certainly shed light on why not all cases of BV result in a positive whiff test.

Quorum sensing (QS) has been identified as a form of communication between bacteria whereby signaling molecules (autoinducing peptides) are released to regulate gene expression in a way that depends on cell density and population needs. Given that regulation of virulence factors is an important component of QS (83), understanding the mechanisms of QS in *Gardnerella* initiated biofilms could identify potential targets for therapeutics against BV-associated biofilms. Autoinducer 2 (AI-2) transporter transcripts are highly abundant in *in vitro G. leopoldii* biofilms but not in their planktonic counterparts (71). *G. vaginalis* also produces the AI-2 signaling molecule required for quorum sensing, which can be inhibited to a degree following benzoyl peroxide (84) as well as by subtilosin (a bacteriocin) treatments (85). Beyond these findings, mechanisms of quorum sensing contributing to *Gardnerella* biofilm formation and persistence are not fully understood. Models to study *Gardnerella*-initiated polymicrobial biofilms are being developed (66, 86), and analysis of those systems through transcriptomic, proteomic, and metabolomic approaches will greatly improve our understanding of QS in these biofilms.

*In vitro*, there is no consensus on which subgroup forms better biofilms (37, 47, 65) and media formulation was shown to affect biofilm formation capabilities of different *Gardnerella* species isolates (58, 87). *G. vaginalis* biofilm formation is enhanced at pH ~5 to 6.5, which is the typical pH range during BV, either due to increased adhesion to the epithelia under these settings and/or reduced interference by lactobacilli (88). In contrast, lower pH (<4.5) typically seen in lactobacilli-dominant states and high pH (>7) result in weak or no *Gardnerella* biofilms, at least *in vitro* (88). It is crucial to understand what factors promote these apparent differences in biofilm formation and why, as well as which best represent conditions found *in vivo* to establish more valid *in vitro* systems to study *Gardnerella* biofilm formation and their effects on the mucosa.

### Vaginolysin.

Vaginolysin, is a cholesterol-dependent hemolysin which lyses susceptible cells upon interaction with the molecule CD59 in a way that forces rapid structural “blebbing” changes in vaginal and cervical epithelial cells (89, 90). Although vaginolysin causes hemolysis of human erythrocytes, bacteremia due to *Gardnerella* is a relatively rare phenomenon and is not a feature of BV (89, 91). The undecapeptide vaginolysin was shown to be orthologous to other Gram-positive cholesterol-dependent cytolysins and appears to function in a human specific manner *in vitro* due to its interactions with the complement glycoprotein CD59 (89). Though vaginolysin can be directly secreted, recent evidence suggests it is also packaged within *G. vaginalis* membrane vesicles, which can be internalized by vaginal epithelial cells (92). Production of vaginolysin as well as other virulence factors in these vesicles is dependent on pH and is not produced under more acidic conditions (such as those found in optimal lactobacilli-dominant vaginal communities) (93). It is unclear how common membrane vesicle formation is among the different *Gardnerella* species and genomospecies. Like other cytolysins, the epithelial response to vaginolysin induces phosphorylation and activation of p38 mitogen-activated protein kinase, and the effect on the epithelium is concentration dependent (89, 94). Not all *Gardnerella* isolates possess vaginolysin genes, as several *G. piotii* isolates and genomospecies 11 seem to lack them, possibly due to purifying selection (59, 65, 95) (Table 1). The gene encoding vaginolysin appears to be exchanged through horizontal gene transfer and is likely not part of the core *Gardnerella* genome as previously thought (36, 59). More than one *Gardnerella* species often cooccur in the vaginal microbiome and during BV (18, 48, 95), therefore differential production of vaginolysin could explain the different roles these species play during the establishment of BV. Vaginolysin expression is upregulated in the planktonic state and reduced in biofilms of *G. leopoldii* (71). Given that vaginolysin is capable of lysing neutrophils (96) and possibly other immune cells such as macrophages, vaginolysin production in the early stages of *Gardnerella* proliferation could represent an early immune evasion mechanism.

Differences in the genetic makeup of vaginolysin exist within the 13 different *Gardnerella* genomospecies, and these have recently been typed into 5 vaginolysin groups — types 1A, 1B, 1C (collectively type 1), type 2, and type 3 (95). Type 1 and 2 are more common among the characterized isolates (Table 1), and type 1A appears to induce higher cytotoxicity even upon reduced CD59 expression compared to type 2. Nonetheless, vaginal epithelial cytokine responses to type 1A and type 2 vaginolysins are similar for the most part (with increased cytokine concentration upon treatment) (95). Vaginolysin, has also been shown to activate proinflammatory signaling in cervical HeLa cells (89), though its effects on vaginal epithelial tissue appear to differ based on apical or basolateral activity likely due to differential CD59 expression (97). BV-associated *Gardnerella* species induced higher cytotoxicity in cervical HeLa cells relative to non-BV isolates, which correlated with increased vaginolysin expression (58, 98). Treatment with L. crispatus reduced *Gardnerella* cytotoxicity *in vitro*, and though vaginolysin expression was also generally reduced in BV-*Gardnerella* isolates, this did not reach statistical significance (98). Specific immune responses to *Gardnerella* vaginolysin have been detected in individuals with BV (99). Antivaginolysin-specific immunoglobulin A (IgA) response has been demonstrated in both healthy and BV samples (100) and may help prevent adverse health outcomes in pregnant women with BV (101). The presence of these higher IgA responses could also be indicative of a low sialidase environment (102), as will be discussed next.

### *Gardnerella* sialidases.

The vaginal mucosa is a protective barrier comprised of sialoglycoproteins called mucins that, among other functions, help prevent adherence of pathogens to the underlying epithelium (103). *Gardnerella* species possess several hydrolytic enzymes such as sialidases that appear to play a significant role in the pathogenesis of BV. Sialidases cleave sialic acid from mucosal sialoglycans to enable bacterial adherence and biofilm formation and allow its use as a nutritional substrate (104). In line with this, the vaginal epithelial glycocalyx appeared to be reduced in BV-positive participants compared to healthy controls. This was further confirmed to be associated with reduced sialylation of surface *N*- and *O*-glycans, and a similar glycan phenotype was produced following treatment with a *Gardnerella* sialidase. Desialylation of these glycans exposes terminal sugars that can then be used for bacterial adherence (105), perhaps via carbohydrate-binding proteins such as lectins which have been predicted in *Gardnerella* (67). Sialidases can also cleave immunoglobulins (secreted sialoglycoproteins) likely as an immune evasion strategy, which may also indirectly increase risk to other mucosal pathogens. In support of this, an association between sialidases and IgA cleavage has been demonstrated (102).

*Gardnerella* isolates possessing sialidase genes have been shown to be associated with BV biofilms (106). This has led to the development of diagnostic tests for BV based on sialidase activity (107–109). In line with this, concentrations of the mucins 5AC and 5B are elevated in cervicovaginal lavage samples with increased bacterial diversity (110). *In vitro*, some *Gardnerella* species (especially subgroup B isolates) cause degradation of vaginal mucins, via anchored cell-associated sialidases and secreted sialidases (104, 111). Secretion of cell wall-anchored sialidases can also occur due to cell turnover or proteolytic cleavage (111, 112). Sialidase activity in *Gardnerella* isolates is primarily attributed to NanH2 and NanH3, whereas NanH1 (sialidase A) genes are also present in isolates that do not show sialidase activity (111). The lack of extracellular sialidase activity associated with NanH1 may be explained by its lack of signal peptide, suggesting it is likely an intracellular enzyme (112). Phenotypic assessments of clinical isolates have shown that sialidase activity varies significantly among the different *Gardnerella* species, with few clade 1 and many clade 2 isolates possessing sialidase activity, whereas clade 4 members do not (19, 38, 65). Sialidase activity is strongest in *G. piotii* and genomospecies 3 (subgroup B) isolates (38, 112), and the presence of sialidase genes in other *Gardnerella* isolates could be due to horizontal gene transfer within the vaginal ecosystem (59). Based on these findings, it can be speculated that colonization by NanH2 and/or NanH3-encoding *Gardnerella* species (especially subgroup B isolates) may be the first step in the establishment of BV biofilms. However, our understanding is incomplete and sialidase is likely only one of several factors in the formation of biofilms and in the pathogenesis of BV.

### Other virulence factors.

Other *Gardnerella* virulence factors have been recognized but are not as well characterized; these include pili, lipases, and siderophores. Pili have been demonstrated in some *Gardnerella* isolates (113, 114), and so has the presence of pili-associated genes (40), though the presence of these genes does not always result in pili expression *in vitro*. Existence of pili in *Gardnerella* species may contribute to virulence on multiple fronts by interacting with the host immune system, enhancing attachment to tissues, and contributing to horizontal transfer of other virulence genes. Iron acquisition strategies, including via siderophore activity has been demonstrated in several *Gardnerella* isolates (115) which may explain increases of *Gardnerella* loads during menses (116). Adequate access to iron is important for the expression of virulence factors (117), and evolution of multiple iron acquisition strategies in some *Gardnerella* isolates can thus promote their survival *in vivo*. The presence of lipases has been demonstrated in several *Gardnerella* isolates (24). Though their role in *Gardnerella* pathogenicity is not well characterized, bacterial lipases can increase nutrient availability, promoting persistence, and may be involved in immune evasion and/or weakening of the epithelial barrier (118).

## IMMUNE RESPONSES TO *GARDNERELLA* SPECIES — FINDINGS FROM *IN VITRO* AND ANIMAL STUDIES

The relationship between BV and mucosal immunity is incompletely understood. Clinically, BV is not characterized by leukocyte infiltration, hence the name “vaginosis” and not “vaginitis.” This shouldn’t be surprising given that BV is recognized as a biofilm condition and the nature of bacterial biofilms to promote a more quiescent response (78). Nonetheless, BV *is* associated with subclinical inflammation characterized by generally high proinflammatory cytokine responses and decreased levels of some chemokines (119–121). Given its predominance in BV biofilms (62), *Gardnerella*-mediated alternations in mucosal immunity could help explain BV persistence and how BV increases the risk for STI and pregnancy complications. This section focuses on findings from *in vitro* and murine studies utilizing single *Gardnerella* isolates to examine how *Gardnerella* species contribute to cytokine production and cell viability (Table S1).

### Immune cell responses to *Gardnerella* species.

Four main types of antigen-presenting cells are found within the vaginal epithelia and lamina propria: CD14^−^ dendritic cells (DCs), CD14^+^ DCs, macrophages, and Langerhans cells (122). DCs sample for pathogens and respond to microbial insults by production of cytokines that contribute to T-cell polarization. The production of these cytokines differs among the DC subsets, such that CD14^−^ DCs favor Th2 activation, and CD14^+^ DCs prime Th1 responses (122). Bertran et al. (123) showed that *G. vaginalis* did not generally induce cytotoxicity or increases in proinflammatory cytokines (TNF-α, IL-12p70, IFN-γ) in monocyte-derived DCs, suggesting a somewhat quiescent response. A different study utilizing the same isolate (as well as *Prevotella* and other BV-associated organisms) did show a significant increase in DC activation markers and increased expression of IL-1β, IL-6, IL-8, IL-12A + IL-12B, and TNF cytokine mRNA (124). However, in the case of *Gardnerella*, the responses across the board were much less pronounced than those in response to *Prevotella* stimulation. Interestingly, at the protein level only IL-8 secretion was significantly increased in response to *Gardnerella*-only stimulation, whereas many other cytokines, including TNF-α, IL-1β, and IL-6, were increased in response to *Prevotella* stimulation (124). The apparent effect on cytokine secretion could be indicative of an altered DC response to *G. vaginalis*. The findings of the latter study are more in line with available literature showing that DC stimulation with cervicovaginal fluid (as opposed to a single microorganism) from BV patients leads to DC activation (125). T-lymphocyte and peripheral blood mononuclear cells challenged with subgroup C isolates increased levels of some proinflammatory cytokines, including IFN-γ and IL-17 (123, 126) but not IP-10 or IFN-α (126). Lymphocyte proliferation seems to occur only at high, BV-consistent *Gardnerella* loads (10^7^ CFU/mL) (123). Studies looking at human and mouse macrophage and monocytic cell lines show increases in IL-12 and TNF-α transcription and secretion, as well as IFN-γ, IL-10, and IL-17 secretion (123, 127) and IL-1β transcription and secretion (127–129), which could be suggestive of increases in Th1, Th17 and T-reg responses. However, these models do not capitulate well the diversity of DC effector and tissue macrophage function found in human vaginal mucosal epithelial tissue. Expression of CCR7 and the costimulatory molecule CD80 were also noted on macrophages following treatment with *G. vaginalis* supernatants, as was the increased production of reactive oxygen species by stimulated macrophages, suggestive of M1 macrophage polarization (127, 128). In other bacterial infections, M1 tissue macrophages are associated with microbial clearance and are activated by the more metabolically active planktonic bacteria (130). It remains unclear whether stimulation of these macrophages with *Gardnerella*-dominated biofilms will reproduce these findings or rather promote an M2 phenotype as reported in other biofilm conditions (130). In monocytic cells, stimulation with lavage samples from individuals with BV resulted in cell activation via TLR2 mediated response but not TLR4 (131). Within macrophages, the resulting signaling cascade leads to activation of NF-κB which then activates the NLRP3 inflammasome. NLRP3 inflammasome leads to caspase-1 production which then cleaves the pro-IL-1β, and pro-IL-18 proteins leading to their upregulated release and eventually causing cell death via pyroptosis (127, 129). This inflammasome activation has been shown to enhance HIV-1 replication which may help explain the increase in HIV-1 susceptibility in individuals with BV. Other immune cell populations, including myeloid derived suppressor cells and innate T-cells (which are present within the epithelia), have been implicated in other biofilm infections (78) but were not studied extensively in the context of *Gardnerella* colonization.

### Cytokine responses of reproductive epithelia and fetal membranes to *Gardnerella* challenge.

Pivotal sites of *Gardnerella* colonization are the stratified squamous epithelium of the vagina and ectocervix, or the simple columnar epithelium of the endocervix and uterus in the case of an ascending infection (132). Therefore, it is not surprising that the majority of *in vitro* and murine studies to date have focused on the immune responses of these sites to *Gardnerella*. Cytokine responses appear to differ both depending on the cell model and *Gardnerella* species tested. For example, results from *in vitro* studies using the same *G. vaginalis* isolates have not been consistent (Table S1). HeLa cell stimulation with a large dose of live bacteria induced significant increases in IL-1β, TNF-α, IL-6, and IL-8 (133), whereas treatment of the same cell line with lysates from the same isolate did not (134). The challenge of cervical and uterine carcinoma cell lines with heat-inactivated *G. vaginalis* isolates also did not cause significant changes in these cytokines (126), though a lower challenge dose was used. Though some studies suggest increased IL-8 production by vaginal and cervical epithelial cells (133, 135–139), BV is not typically associated with an increase in neutrophil counts (140). However, in murine models, inoculation with *G. vaginalis* does increase vaginal myeloperoxidase activity (141). One possible explanation for this is lysis of neutrophils by vaginolysin in some settings. This IL-8 response also seems to differ depending on the *Gardnerella* species used, as do other chemokine responses (97). In the lower female genital tract, the vaginal epithelium has a different PRR profile compared to endocervical cells, and to immune cells in the basement membrane which may explain differences in the immune responses to the same pathogen (142). For example, increases in IP-10 production were present following endocervical *G. vaginalis* challenge but not vaginal or ectocervical challenge (143) though treatment with cell free supernatants from a related isolate did induce IP-10 secretion in ectocervical cells (137). In contrast, in response to *G. leopoldii* isolate treatment, secreted IP-10 was reduced in both apical and basolateral ectocervical-vaginal compartments (97). No change was observed in response to *G. vaginalis* isolates (138, 143), and an increase was observed in response to an uncharacterized clinical isolate in vaginal epithelial cells (139). Whether these responses are consistent *in vivo* or across models, and whether regulation of these occurs at the transcriptional, translational, or secretory level is unclear. Reduced chemokine responses have been described in response to anaerobic oral infections in which *Prevotella* species are prominent, and with respect to IP-10 and related CXCR3 chemokines in response to intracellular pathogens (144, 145). Reversal of this CXCR3 chemokine response seems to be a marker of successful BV treatment, though a causal link is yet to be elucidated (146). IP-10 and related CXCR3 chemokines have effects on Th1 cells, CD8^+^ T cells, and other lymphocytes, including natural killer cells and γδ T cells (147), which are understudied in the context of BV. Several hypotheses could help explain how *Gardnerella* and other BV-associated species exert their effects on mucosal immunity, including via short-chain fatty acid production (148), stimulation of different host signaling pathways, or even production of enzymes and toxins capable of degrading cytokines and mucosal components as reported in other bacterial and biofilm conditions.

BV is associated with increased risk to pregnancy complications, including preterm birth (11), possibly due to ascending infection and/or stimulation of inflammation (149). Treatment of fetal membranes with *Gardnerella* isolates (mostly *G. vaginalis*) promoted secretion of the proinflammatory cytokines IL-6, TNF-α, and IL-1β (mostly from the choriodecidual compartment) (136, 150–152), and the immune-modulatory cytokine IL-10 (153–155). Production of beta-defensins such as HBD-1 also increased following *Gardnerella* stimulation (150). With respect to PRR signaling, TLR-2 and TLR-7 were somewhat upregulated in fetal membranes in response to *G. vaginalis* although this was not statistically significant (156). *Gardnerella* has been detected more frequently in the cervix of women who underwent premature rupture of membranes with evidence of intraamniotic microbial invasion but without accompanying intraamniotic inflammation (157). In support of this, the increase in IL-6 in response to *Gardnerella* challenge of fetal membranes was accompanied by increased production of gp130 and mIL-6R (151). This suggests that IL-6 response to *Gardnerella* occurs through the gp130 and mIL-6 membrane receptor which induces antiinflammatory action (151). This again supports the hypothesis that as biofilms *Gardnerella* species either do not induce or somehow suppress some inflammatory responses. To add to this, *Gardnerella* biofilms within the endometrium and fallopian tubes have been reported *in vivo* and presence of these biofilms was more likely in pregnant and BV positive individuals (132). Stimulation of *in vitro* 3-dimensional endometrial epithelial cell models with *G. piotii* isolates did not elicit any significant cytokine responses, though one of the isolates did slightly increase MIP-3α secretion (158). *Gardnerella* has also been proposed to enhance ascension of perhaps more pathogenic organisms such as group B streptococci or mycoplasmas into fetal membranes. This was supported in a mouse model, where coinoculation resulted in invasive infection of placental tissue accompanied by placental histopathology (152). In a separate study, *G. vaginalis* was shown to induce IL-8, IL-6, IL-10, and IL-1β expression in the cervicovaginal space of pregnant mice, but reduced expression of the proinflammatory cytokine TNF-α relative to negative controls. *G. vaginalis*-induced cervical remodeling/breakdown was also proposed in this model due to increased expression of soluble E-cadherin and Tff-1 (136), possibly due to microRNA regulation. MicroRNAs and gene regulation by *Gardnerella* metabolites have also been proposed as potential mediators of the link between BV and pregnancy complications. In support of this hypothesis, *G. vaginalis* supernatant treatment of ectocervical cells resulted in increased expression of miRNAs associated with epithelial barrier disruption and short gestation (137), an effect that was mostly reversed upon treatment with optimal lactobacilli supernatants. Nonetheless, given that pregnancy can alter TLR expression and mucosal antimicrobial properties, validation of these miRNA findings is necessary.

## SUMMARY OF KNOWLEDGE GAPS AND CHALLENGES

The role of *Gardnerella* in reproductive health remains incompletely understood. On the one hand, *Gardnerella* is a core component of BV biofilms, and on the other, it has also been isolated from healthy individuals. Advances to resolve *Gardnerella* strains into pathogenic and nonpathogenic categories have been proven challenging. Recently, the *Gardnerella* genus was emended to include several different species and genomospecies, which do appear to possess different virulence potentials. The regulation of these virulence factors and their effects on the mucosal epithelia remain ambiguous and are currently being investigated through systems biology approaches and *in vitro* models. Given that the biofilm form of *Gardnerella* appears to be the most problematic for reproductive health, identification of bacterial components involved in adhesion and biofilm formation will be beneficial for the design of more targeted interventions. Perhaps the most significant gaps in knowledge are with respect to *Gardnerella* effects on cervicovaginal mucosal immune responses and how those differ among the different genomospecies and the biofilm and planktonic growth states. We reviewed findings from *in vitro* and murine-controlled *Gardnerella* challenge studies which mostly used *G. vaginalis* isolates and show some upregulation in proinflammatory responses in some models but not in others. With respect to some chemokines such as IP-10, *in vitro* studies seem to show discrepant results in response to *Gardnerella*. Whether this is due to model and/or *Gardnerella* species differences is unclear. An area remaining completely uncharacterized with respect to *Gardnerella* immunobiology is the interactions with myeloid-derived suppressor cells as well as intraepithelial lymphocytes, which have been implicated in other biofilm conditions. Further investigation of these immune responses when combined with consideration of *Gardnerella* heterogeneity will greatly enhance our understanding of *Gardnerella* species contributions to reproductive health.
