# Supplementary material for: Gardnerella Revisited: Species Heterogeneity, Virulence Factors, Mucosal Immune Responses, and Contributions to Bacterial Vaginosis
Source: Infect Immun. 2023 Apr 18;91(5):e00390-22. doi: 10.1128/iai.00390-22 (PMC10187134; doi:10.1128/iai.00390-22)
Supplement: Supplemental file 1 — Table S1. Download iai.00390-22-s0001.pdf, PDF file, 0.4 MB [file iai.00390-22-s0001.pdf]

Supplementary Table 1. Summary of cytokine and cytotoxicity findings from in-vitro and murine *Gardnerella* challenge studies assessing cytokine responses. Publication search included PubMed search criteria of "Gardnerella" AND "Cytokines" NOT "Review".

Tested isolates	Subgroup/ Clade	Bacterial dose(s)	Cell or Animal Model	Cytokines tested	Significant Cytokine Signatures (secreted)	Significant Cytokine Signatures (mRNA)	Immune Activation	Cytotoxicity/ Viability	Ref.	Year
**Immune cells**										
ATCC 14018	C/1	MOI of 5-10 (live bacteria)	PMA differentiated human THP-1 (Macrophage like cells)	TNF-α, IL-1β, IL-18	↑ TNF-α, IL-1β, IL-18 (24-hour treatment)	N/A	N/A	Slight reduction in viability at 24 hours, likely due to pyroptosis	(1)	2014
ATCC 14018	C/1	10^3-10^7 CFU/mL	PHA induced T-cells	IFN-γ, IL-4, IL-17A, IL-10, IL-12p70, and TNF-α	↑ TNF-α, IL-12p70, IFN-γ, IL-17A, IL-10 (more pronounced at highest bacterial dose)	N/A	Increased proliferation at high doses	N/A	(2)	2016
ATCC 14018	C/1	10^3-10^7 CFU/mL	PBMC derived Dendritic cells	IL-10, TNF-α, IFN-γ, and IL-12p70	↑IL-10 (marginal) - at highest bacterial dose	N/A	low level maturation (reduced MR CD206)	None detected at the tested doses (10^3-10^7 CFU/mL)	(2)	2016
ATCC 14018 (DSM 4944)	C/1	MOI of 1 + 10	PBMC derived Dendritic cells	IL-1β, IL-8, IL-12A + IL-12B (IL12p70), IL-10, IL-6, TNF (TNF-α)	↑ IL-8 (24-hour treatment)	↑IL-1β, IL-6, IL-8, IL-12A + IL-12B, TNF (6-hour treatment)	Increased expression of CD80, CD83, CD86 (more pronounced at highest bacterial dose)	N/A	(3)	2020

Tested isolates	Subgroup/ Clade	Bacterial dose(s)	Cell or Animal Model	Cytokines tested	Significant Cytokine Signatures (secreted)	Significant Cytokine Signatures (mRNA)	Immune Activation	Cytotoxicity/ Viability	Ref.	Year
**Immune cells**										
KCTC5096 (ATCC 14018)	C/1	MOI of 10	PMA differentiated human THP-1 (Macrophage like cells)	TNF-α, IL-1β, IL-18	↑ TNF-α, IL-1β, IL-18	N/A	Induced production of ROS, and NLRP3, and caspase-1 protein expression, and apoptosis associated speck like protein production	Significantly reduced viability	(4)	2021
KCTC5096 (ATCC 14018)	C/1	MOI of 10	Mouse peritoneal primary macrophages	TNF-α, IL-1β, IL-18	↑ TNF-α, IL-1β, IL-18	N/A	Induced production of ROS, and NLRP3, and caspase 1protein expression, and apoptosis associated speck like protein production	Significantly reduced viability	(4)	2021

Tested isolates	Subgroup/ Clade	Bacterial dose(s)	Cell or Animal Model	Cytokines tested	Significant Cytokine Signatures (secreted)	Significant Cytokine Signatures (mRNA)	Immune Activation	Cytotoxicity/ Viability	Ref.	Year
**Immune cells**										
315-A, ATCC 49145, ATCC 14019 and ATCC 14018 (pooled lysates)	C/1	N/A	PBMCs	IFN-γ, IL-17, IL-10	None compared to unstimulated control. Significant decrease in IFN-γ relative to lactobacilli	N/A	N/A	N/A	(5)	2021
315-A, ATCC 49145, ATCC 14019 and ATCC 14018	C/1	MOI of 50	PBMCs	IFN-γ, IL-17, IL-10, IL-6, IP-10, IFN-α, IL-4, IL-8	↑ IFN-γ, IL-17, IL-10, IL-6 (5 days)	N/A	N/A	N/A	(5)	2021
ATCC 14018	C/1	Cell free supernatants (5%+10%)	human THP-1 (PMA stimulated)	IL-12, IL-1β, IP-10, TNF-α, IL-6	N/A	↑IL-12, IL-1β, CXCL10, TNF-α	↑ CCR7 transcription, and detection of surface CD80	N/A	(6)	2021
ATCC 14018	C/1	Cell free supernatants (5%+10%)	mouse RAW264.7	IL-12, IL-1β, IP-10, TNF-α, IL-6	N/A	↑ IL-12, IL-6, TNF-α, IL-1β	↑ CCR7 transcription, and detection of surface CD80	N/A	(6)	2021

Tested isolates	Subgroup/ Clade	Bacterial dose(s)	Cell or Animal Model	Cytokines tested	Significant Cytokine Signatures (secreted)	Significant Cytokine Signatures (mRNA)	Immune Activation	Cytotoxicity /Viability	Ref.	Year
**Reproductive cells**										
ATCC 14018 (lysate)	C/1	50, 500 or 5000 ng/mL lysates	HeLa	IL-6, IL-1α, TNF-α, IL-8	None	N/A	N/A	N/A	(7)	1999
Uncharacterized isolate (lysate)	-	50, 500 or 5000 ng/mL lysates	HeLa	IL-6, IL-1α, TNF-α, IL-8	None	N/A	N/A	N/A	(7)	1999
Clinical isolate	-	OD620 of 1.0, 0.1, 0.01, or 0.001	Organotypic vaginal epithelium with dendritic cells (apical surface was challenged)	IL-8, IL-1	N/A	None	N/A	N/A	(8)	2006
AmMS 117 (ATCC 49145)	C/1	MOI of 0.1, 1, and 10	VK2	IL-6, IL-8, IL-1β, TNF-a, IL-10	↑ IL-6 (at highest dose)	N/A	N/A	N/A	(9)	2008
KCTC5096 (ATCC 14018)	C/1	$6x10^6$ CFU/mouse or $1.2x10^5$ CFU/mouse	β-estradiol-3-benzoate immunosuppressed mouse vagina	TNF-α, IL-6, IL-10, IL-1β	↑ TNF-α, IL-6, IL-1β ↓ IL-10	N/A	Increased transcription of COX-2, iNOS, and NF-κB	N/A	(10, 11)	2011

Tested isolates	Subgroup/ Clade	Bacterial dose(s)	Cell or Animal Model	Cytokines tested	Significant Cytokine Signatures (secreted)	Significant Cytokine Signatures (mRNA)	Immune Activation	Cytotoxicity /Viability	Ref.	Year
**Reproductive cells**										
ATCC 49145	C/1	MOI of 15 (average)	End1/E6E7	PDGF, IL-1β, IL-1RA, IL-6, IL-7, IL-8, IL-10, IL-12p70, IL-13, G-CSF, IFN-γ, IP-10, MIP-1β, RANTES, VEGF, Gro-α, IL-1α, LIF, M-CSF, MIF, SDF-1α	↑ PDGF, IL-6, G-CSF, IP-10	N/A	N/A	N/A	(12)	2012
ATCC 49145	C/1	MOI of 15 (average)	Ect1/E6E7	PDGF, IL-1β, IL-1RA, IL-6, IL-7, IL-8, IL-10, IL-12p70, IL-13, G-CSF, IFN-γ, IP-10, MIP-1β, RANTES, VEGF, Gro-α, IL-1α, LIF, M-CSF, MIF, SDF-1α	↑ IL-6, IL-7, G-CSF	N/A	N/A	N/A	(12)	2012

Tested isolates	Subgroup/ Clade	Bacterial dose(s)	Cell or Animal Model	Cytokines tested	Significant Cytokine Signatures (secreted)	Significant Cytokine Signatures (mRNA)	Immune Activation	Cytotoxicity /Viability	Ref.	Year
**Reproductive cells**										
ATCC 49145	C/1	MOI of 15 (average)	VK2	PDGF, IL-1β, IL-1RA, IL-6, IL-7, IL-8, IL-10, IL-12p70, IL-13, G-CSF, IFN-γ, IP-10, MIP-1β, RANTES, VEGF, Gro-α, IL-1α, LIF, M-CSF, MIF, SDF-1α	↑ PDGF, IL-6, IL-7, G-CSF	N/A	N/A	N/A	(12)	2012
Uncharacterized isolate	-	$2x10^6$ CFU/well	Immortalized human vaginal epithelial cells (HVECs)	IL-8	↑ IL-8	N/A	N/A	N/A	(13)	2013
JCP8066	B/2	MOI of 10	3D human endometrial epithelial cell (HEC-1-A) aggregates	IL-6 (protein only), IL-8, TNF (TNF-α), MIP-3α, IL-1β (mRNA only)	↑ MIP-3α	None	N/A	low level <5%	(14)	2017

Tested isolates	Subgroup/ Clade	Bacterial dose(s)	Cell or Animal Model	Cytokines tested	Significant Cytokine Signatures (secreted)	Significant Cytokine Signatures (mRNA)	Immune Activation	Cytotoxicity /Viability	Ref.	Year
**Reproductive cells**										
JCP8151B	B/2	MOI of 10	3D human endometrial epithelial cell (HEC-1-A) aggregates	IL-6 (protein only), IL-8, TNF (TNF-α), MIP-3α, IL-1β (mRNA only)	None	None	N/A	low level <5%	(14)	2017
KCTC5096 (ATCC 14018)	C/1	$1x10^8$ CFU/mouse	β-estradiol-3-benzoate immunosuppressed mouse vagina	TNF-α, IL-1β, IL-10, IL-17A	↑ IL-17A, TNF-α, IL-1β ↓ IL-10	N/A	Reduced Foxp3 and increased RORγt transcription in vaginal tissue	N/A	(15)	2017
ATCC 14018	C/1	MOI of 500	HeLa	IL-6, IL-8, IL-1β, TNF-α, IL-10	↑ IL-1β, TNF-α, IL-6, IL-8	↑IL-8 (did not measure mRNA of other cytokines)	Induced NF-κB activation	N/A	(16)	2018
ATCC 14019	C/1	$2.5x10^7$ CFU	Pregnant CD-1 mice (vaginal challenge - cytokines measured in cervicovaginal fluid/cervical tissue)	IL-6 (protein) and IL-1β, TNF, IL-10, IL-8 (mRNA)	↑ IL-6	↑ IL-8, IL-10, IL-1β	N/A	N/A	(17)	2018

Tested isolates	Subgroup/ Clade	Bacterial dose(s)	Cell or Animal Model	Cytokines tested	Significant Cytokine Signatures (secreted)	Significant Cytokine Signatures (mRNA)	Immune Activation	Cytotoxicity /Viability	Ref.	Year
**Reproductive cells**										
Clinical sialidase +, Strep resistant isolate	-	$5x10^7$ CFU/mouse	β-estradiol treated C57BL/6 mouse vagina	TNF-α, IL-6, IL-10, IL-1β	None	N/A	N/A	N/A	(18)	2018
ATCC 14018 (JCM11026)	C/1	Cell free supernatants (10%)	Ect1/E6E7	IL-6, IL-8, TGF-α, G-CSF, Flt-3L, GM-CSF,GRO1, IL-10, IL-12p40, MDC, PDGF-AA, PDGF-AB/BB, IL-15, IL-1RA, IL-1α, IL-1β, IL-7, IP-10, RANTES, TNF-α, VEGF	↑ TGF-α, G-CSF, GM-CSF, GRO1, PDGF-AA, IL-15, IL-1α, IL-1β, IL-6, IL-7, IL-8, IP-10, RANTES, TNF-α, VEGF	N/A	N/A	Increased permeability for both Ect1 and End1 cells	(19)	2019

Tested isolates	Subgroup/ Clade	Bacterial dose(s)	Cell or Animal Model	Cytokines tested	Significant Cytokine Signatures (secreted)	Significant Cytokine Signatures (mRNA)	Immune Activation	Cytotoxicity /Viability	Ref.	Year
**Reproductive cells**										
AMD	A/4	10^5 CFU	Apical surface of partial thickness EpiVaginal tissue (derived from primary vaginal-ectocervical cells)	IL-1ra, IL-2, IL-4, IL-6, IL-7, IL-8, IL-9, IL-12, IL-15, IL-17A, Eotaxin, G-CSF, GM-CSF, IFN-γ, IP-10, MCP-1, MIP-1α, PDGF-bb, MIP-1β, RANTES, TNF-α, VEGF, IL-1β	↑ IL-1ra, IFN-γ, PDGF-bb ↓ IL-6, IL-8, IL-9, GM-CSF, MCP-1, MIP-1β, RANTES, VEGF, (IP-10 - trending)	N/A	N/A	Increased cytotoxicity	(20)	2019
AMD	A/4	10^5 CFU	Basolateral surface of partial thickness EpiVaginal tissue (derived from primary vaginal-ectocervical cells)	IL-1ra, IL-2, IL-4, IL-6, IL-7, IL-8, IL-9, IL-12, IL-15, IL-17A, Eotaxin, G-CSF, GM-CSF, IFN-γ, IP-10, MCP-1, MIP-1α, PDGF-bb, MIP-1β, RANTES, TNF-α, VEGF, IL-1β	↑ IL-1ra, Eotaxin, IL-1β, IL-12 ↓ IP-10	N/A	N/A	Increased cytotoxicity, more pronounced in basolateral side than apical	(20)	2019

Tested isolates	Subgroup/ Clade	Bacterial dose(s)	Cell or Animal Model	Cytokines tested	Significant Cytokine Signatures (secreted)	Significant Cytokine Signatures (mRNA)	Immune Activation	Cytotoxicity /Viability	Ref.	Year
**Reproductive cells**										
KCTC5096 (ATCC 14018)	C/1	1x10^6 CFU/mouse	β-estradiol-3-benzoate immunosuppressed mouse (C57BL/6) vagina	TNF-α, IL-10	↑ TNF-α ↓ IL-10	↑ TNF-α	Increase in COX-2, iNOS, and p-p65 (NF-KB activation) expression, also noted an increase in myeloperoxidase activity	N/A	(21)	2019
ATCC 14018	C/1	1x10^7 CFU/mL	VK2	IL-6, IL-8, IL-1α, IL-1β, IP-10, MIP-3α, MIP-1α, MIP-1β, IL-1RA	↑ IL-1α, IL-1β, IL-6, IL-8, MIP-3α, MIP-1β, MIP-1α (compared to cells only)	N/A	N/A	N/A	(22)	2020
JCP8151B	B/2	MOI of 10	Immortalized human 3D endocervical epithelia cell (A2EN) aggregates	IL36-γ, IL-8, MIP-3α, TNF	N/A	↑IL36-γ, TNF, MIP-3α	N/A	N/A	(23)	2020
315-A	C/1	MOI of 50	SiHa and CaSki	IL-1β, TNF-α, IL-8	None compared to unstimulated control	N/A	N/A	Reduced viability - dose dependent	(5)	2021
ATCC 49145	C/1	MOI of 50	SiHa and CaSki	IL-1β, TNF-α, IL-8	None compared to unstimulated control	N/A	N/A	Reduced viability - dose dependent	(5)	2021

Tested isolates	Subgroup/ Clade	Bacterial dose(s)	Cell or Animal Model	Cytokines tested	Significant Cytokine Signatures (secreted)	Significant Cytokine Signatures (mRNA)	Immune Activation	Cytotoxicity /Viability	Ref.	Year
**Reproductive cells**										
ATCC 14018	C/1	MOI of 50	SiHa and CaSki	IL-1β, TNF-α, IL-8	None compared to unstimulated control	N/A	N/A	Reduced viability - dose dependent - not significant for CaSki cells	(5)	2021
ATCC 14019	C/1	MOI of 50	SiHa and CaSki	IL-1β, TNF-α, IL-8	None compared to unstimulated control	N/A	N/A	Reduced viability - dose dependent	(5)	2021
JCP8151B	B/2	MOI of 10-40	Immortalized human 3D endocervical epithelia cell (A2EN) aggregates	IL-1α, IL-1β, IL-6, MIF, TNF-α, TRAIL, MCP-1, MIP-1α, MIP-1β, RANTES, MIP-3α, IL-8, IP-10, TGF-α, VEGF	↑ IL-6, TNF-α	N/A	N/A	Increased concentrations of MMP-9, and reduced MUC1	(24)	2021
Clinical isolate	-	Not stated	VK2	RANTES, MIP-3α, MIP-1β, IP-10, IL-8, IL-1β	↑ IP-10, IL-8, MIP-3α, RANTES	N/A	N/A	N/A	(25)	2021

Tested isolates	Subgroup/ Clade	Bacterial dose(s)	Cell or Animal Model	Cytokines tested	Significant Cytokine Signatures (secreted)	Significant Cytokine Signatures (mRNA)	Immune Activation	Cytotoxicity /Viability	Ref.	Year
**Reproductive cells**										
ATCC 49145	C/1	$2x10^6$ CFU	BALB/c mouse vagina	TNF-α, IL-6, IL-1β	None	None	Also looked at blood cell populations and saw no significant changes	N/A	(26)	2021
KCTC5096 (ATCC 14018)	C/1	$5x10^6$ CFU/mouse	β-estradiol-3-benzoate immunosuppressed mouse (C57BL/6) vagina	TNF-α, IL-6, IL-1β	↑ TNF-α, IL-6, IL-1β	N/A	Increased myeloperoxidase activity and nitric oxide production	Increased vaginal epithelial exfoliation	(27)	2022
**Fetal Membranes**										
Clinical isolate	-	$1x10^6$ CFU	Human chrioamniotic membrane explant transwell culture	TNF-α, IL-6, IL-1β	Choriodecidual compartment: ↑ IL-1β, IL-6 Amniotic compartment: ↑ IL-6	N/A	Choriodecidual compartment: ↑ HBD-1 (only when also applied to amnion), HBD-2, HBD-3 Amniotic compartment: ↑ HBD-3	N/A	(28)	2012

Tested isolates	Subgroup/ Clade	Bacterial dose(s)	Cell or Animal Model	Cytokines tested	Significant Cytokine Signatures (secreted)	Significant Cytokine Signatures (mRNA)	Immune Activation	Cytotoxicity /Viability	Ref.	Year
**Fetal Membranes**										
ATCC 49145	C/1	10^7 CFU	Human fetal membranes	IL-1β, TNF-α, IL-10, IL-8, IL-2	↑ IL-1β, TNF-α, IL-10, IL-8	N/A	N/A	N/A	(29)	2012
Not stated	-	$1x10^6$ CFU	Human chrioamniotic membrane explant transwell culture	IL-10	↑ IL-10	N/A	N/A	N/A	(30)	2013
ATCC 49145	C/1	10^6 CFU	Human fetal membranes	IL-1β, IL-8, TNF-α, GM-CSF, IL-4, IL-10, IL-13	↑ IL-1β, IL-8, TNF-α, GM-CSF, IL-10	N/A	No changes in soluble receptors sIL-1R2, sTNFR-1, trending increase in sTNFR-2	N/A	(31)	2016
ATCC 14019	C/1	$2.5x10^7$ CFU	Pregnant CD-1 mice (vaginal challenge - cytokines measured in amniotic fluid)	IL-6	↑ IL-6	N/A	N/A	N/A	(17)	2018

Tested isolates	Subgroup/ Clade	Bacterial dose(s)	Cell or Animal Model	Cytokines tested	Significant Cytokine Signatures (secreted)	Significant Cytokine Signatures (mRNA)	Immune Activation	Cytotoxicity /Viability	Ref.	Year
**Fetal Membranes**										
ATCC 49145	C/1	10^6 CFU	Human fetal membranes	IL-6 (protein only) and the soluble receptors sIL-6R (protein only), sgp130, and membrane receptor mIL-6R (mRNA)	↑ IL-6	↓ sgp-130, mIL-6R (though histological staining showed higher mIL-6R on chorion and amnion membranes)	N/A	N/A	(32)	2018
JCP8151B-SmR	B/2	~10^8 CFU	Pregnant C57BL/6NCR mice (vaginal challenge - cytokines measured in placental homogenates)	G-CSF, MIP-1α, IL-1β, Eotaxin, IFN-γ, IL-12p70, KC, IL-2, IL-6, MIP-1β, RANTES	↑ IL-2, IL-6, MIP-1β	N/A	N/A	N/A	(33)	2021

References:

1. Vick EJ, Park HS, Huff KA, Brooks KM, Farone AL, Farone MB. 2014. Gardnerella vaginalis triggers NLRP3 inflammasome recruitment in THP-1 monocytes. J Reprod Immunol 106:67-75.
2. Bertran T, Brachet P, Vareille-Delarbre M, Falenta J, Dosgilbert A, Vasson MP, Forestier C, Tridon A, Evrard B. 2016. Slight Pro-Inflammatory Immunomodulation Properties of Dendritic Cells by Gardnerella vaginalis: The "Invisible Man" of Bacterial Vaginosis? J Immunol Res 2016:9747480.
3. van Teijlingen NH, Helgers LC, Zijlstra-Willems EM, van Hamme JL, Ribeiro CMS, Strijbis K, Geijtenbeek TBH. 2020. Vaginal dysbiosis associated-bacteria Megasphaera elsdenii and Prevotella timonensis induce immune activation via dendritic cells. J Reprod Immunol 138:103085.

4. Xiang N, Yin T, Chen T. 2021. Gardnerella vaginalis induces NLRP3 inflammasome-mediated pyroptosis in macrophages and THP-1 monocytes. Exp Ther Med 22:1174.
5. Nicolo S, Tanturli M, Mattiuz G, Antonelli A, Baccani I, Bonaiuto C, Baldi S, Nannini G, Menicatti M, Bartolucci G, Rossolini GM, Amedei A, Torcia MG. 2021. Vaginal Lactobacilli and Vaginal Dysbiosis-Associated Bacteria Differently Affect Cervical Epithelial and Immune Homeostasis and Anti-Viral Defenses. Int J Mol Sci 22:6487.
6. Liu CW, Su BC, Chen JY. 2021. Tilapia Piscidin 4 (TP4) Reprograms M1 Macrophages to M2 Phenotypes in Cell Models of Gardnerella vaginalis - Induced Vaginosis. Front Immunol 12:773013.
7. Hashemi FB, Ghassemi M, Roebuck KA, Spear GT. 1999. Activation of human immunodeficiency virus type 1 expression by Gardnerella vaginalis. J Infect Dis 179:924-30.
8. Valore EV, Wiley DJ, Ganz T. 2006. Reversible deficiency of antimicrobial polypeptides in bacterial vaginosis. Infect Immun 74:5693-702.
9. Libby EK, Pascal KE, Mordechai E, Adelson ME, Trama JP. 2008. Atopobium vaginae triggers an innate immune response in an in vitro model of bacterial vaginosis. Microbes Infect 10:439-46.
10. Joo HM, Hyun YJ, Myoung KS, Ahn YT, Lee JH, Huh CS, Han MJ, Kim DH. 2011. Lactobacillus johnsonii HY7042 ameliorates Gardnerella vaginalis-induced vaginosis by killing Gardnerella vaginalis and inhibiting NF-κB activation. Int Immunopharmacol 11:1758-65.
11. Trinh HT, Lee IA, Hyun YJ, Kim DH. 2011. Artemisia princeps Pamp. Essential oil and its constituents eucalyptol and α-terpineol ameliorate bacterial vaginosis and vulvovaginal candidiasis in mice by inhibiting bacterial growth and NF-κB activation. Planta Med 77:1996-2002.
12. Eade CR, Diaz C, Wood MP, Anastos K, Patterson BK, Gupta P, Cole AL, Cole AM. 2012. Identification and characterization of bacterial vaginosis-associated pathogens using a comprehensive cervical-vaginal epithelial coculture assay. PLoS One 7:e50106.
13. Brosnahan AJ, Merriman JA, Salgado-Pabón W, Ford B, Schlievert PM. 2013. Enterococcus faecalis inhibits superantigen toxic shock syndrome toxin-1-induced interleukin-8 from human vaginal epithelial cells through tetramic acids. PLoS One 8:e61255.
14. Łaniewski P, Gomez A, Hire G, So M, Herbst-Kralovetz MM. 2017. Human Three-Dimensional Endometrial Epithelial Cell Model To Study Host Interactions with Vaginal Bacteria and Neisseria gonorrhoeae. Infect Immun 85:e01049-16.
15. Jang SE, Jeong JJ, Choi SY, Kim H, Han MJ, Kim DH. 2017. Lactobacillus rhamnosus HN001 and Lactobacillus acidophilus La-14 Attenuate Gardnerella vaginalis-Infected Bacterial Vaginosis in Mice. Nutrients 9:531.
16. Santos CMA, Pires MCV, Leão TL, Silva AKS, Miranda LS, Martins FS, Silva AM, Nicoli JR. 2018. Anti-inflammatory effect of two Lactobacillus strains during infection with Gardnerella vaginalis and Candida albicans in a HeLa cell culture model. Microbiology (Reading) 164:349-358.

17. Sierra LJ, Brown AG, Barila GO, Anton L, Barnum CE, Shetye SS, Soslowsky LJ, Elovitz MA. 2018. Colonization of the cervicovaginal space with Gardnerella vaginalis leads to local inflammation and cervical remodeling in pregnant mice. PLoS One 13:e0191524.
18. Sabbatini S, Monari C, Ballet N, Mosci P, Decherf AC, Pélerin F, Perito S, Scarpelli P, Vecchiarelli A. 2018. Saccharomyces cerevisiae-based probiotic as novel anti-microbial agent for therapy of bacterial vaginosis. Virulence 9:954-966.
19. Anton L, Sierra LJ, DeVine A, Barila G, Heiser L, Brown AG, Elovitz MA. 2018. Common Cervicovaginal Microbial Supernatants Alter Cervical Epithelial Function: Mechanisms by Which Lactobacillus crispatus Contributes to Cervical Health. Front Microbiol 9:2181.
20. Garcia EM, Kraskauskiene V, Koblinski JE, Jefferson KK. 2019. Interaction of Gardnerella vaginalis and Vaginolysin with the Apical versus Basolateral Face of a Three-Dimensional Model of Vaginal Epithelium. Infect Immun 87:e00646-18.
21. Kim DE, Kim JK, Han SK, Jang SE, Han MJ, Kim DH. 2019. Lactobacillus plantarum NK3 and Bifidobacterium longum NK49 Alleviate Bacterial Vaginosis and Osteoporosis in Mice by Suppressing NF-κB-Linked TNF-α Expression. J Med Food 22:1022-1031.
22. Manhanzva MT, Abrahams AG, Gamieldien H, Froissart R, Jaspan H, Jaumdally SZ, Barnabas SL, Dabee S, Bekker LG, Gray G, Passmore JS, Masson L. 2020. Inflammatory and antimicrobial properties differ between vaginal Lactobacillus isolates from South African women with non-optimal versus optimal microbiota. Sci Rep 10:6196.
23. Gardner JK, Laniewski P, Knight A, Haddad LB, Swaims-Kohlmeier A, Herbst-Kralovetz MM. 2020. Interleukin-36gamma Is Elevated in Cervicovaginal Epithelial Cells in Women With Bacterial Vaginosis and In Vitro After Infection With Microbes Associated With Bacterial Vaginosis. J Infect Dis 221:983-988.
24. Łaniewski P, Herbst-Kralovetz MM. 2021. Bacterial vaginosis and health-associated bacteria modulate the immunometabolic landscape in 3D model of human cervix. NPJ Biofilms Microbiomes 7:88.
25. Fichorova RN, DeLong AK, Cu-Uvin S, King CC, Jamieson DJ, Klein RS, Sobel JD, Vlahov D, Yamamoto HS, Mayer KH. 2021. Protozoan-Viral-Bacterial Co-Infections Alter Galectin Levels and Associated Immunity Mediators in the Female Genital Tract. Front Cell Infect Microbiol 11:649940.
26. Selis NN, Oliveira HBM, Souza CLS, Almeida JB, Andrade YMFS, Silva LSC, Romano CC, Rezende RP, Yatsuda R, Uetanabaro APT, Marques LM. 2021. Lactobacillus plantarum Lp62 exerts probiotic effects against Gardnerella vaginalis ATCC 49154 in bacterial vaginosis. Lett Appl Microbiol 73:579-589.
27. Choi SI, Won G, Kim Y, Kang CH, Kim GH. 2022. Lactobacilli Strain Mixture Alleviates Bacterial Vaginosis through Antibacterial and Antagonistic Activity in *Gardnerella vaginalis*-Infected C57BL/6 Mice. Microorganisms 10:471.
28. Zaga-Clavellina V, Martha RV, Flores-Espinosa P. 2012. In vitro secretion profile of pro-inflammatory cytokines IL-1β, TNF-α, IL-6, and of human beta-defensins (HBD)-1, HBD-2, and HBD-3 from human chorioamniotic membranes after selective stimulation with Gardnerella vaginalis. Am J Reprod Immunol 67:34-43.

29. Peltier MR, Drobek CO, Bhat G, Saade G, Fortunato SJ, Menon R. 2012. Amniotic fluid and maternal race influence responsiveness of fetal membranes to bacteria. J Reprod Immunol 96:68-78.
30. Zaga-Clavellina V, Flores-Espinosa P, Pineda-Torres M, Sosa-González I, Vega-Sánchez R, Estrada-Gutierrez G, Espejel-Núñez A, Flores-Pliego A, Maida-Claros R, Estrada-Juárez H, Chávez-Mendoza A. 2014. Tissue-specific IL-10 secretion profile from term human fetal membranes stimulated with pathogenic microorganisms associated with preterm labor in a two-compartment tissue culture system. J Matern Fetal Neonatal Med 27:1320-7.
31. Noda-Nicolau NM, Polettini J, Peltier MR, da Silva MG, Menon R. 2016. Combinations and loads of bacteria affect the cytokine production by fetal membranes: An in vitro study. Am J Reprod Immunol 76:504-511.
32. Noda-Nicolau NM, Polettini J, da Silva MG, Peltier MR, Menon R. 2018. Polybacterial stimulation suggests discrete IL-6/IL-6R signaling in human fetal membranes: Potential implications on IL-6 bioactivity. J Reprod Immunol 126:60-68.
33. Gilbert NM, Foster LR, Cao B, Yin Y, Mysorekar IU, Lewis AL. 2021. Gardnerella vaginalis promotes group B Streptococcus vaginal colonization, enabling ascending uteroplacental infection in pregnant mice. Am J Obstet Gynecol 224:530.e1-530.e17.
